# Core Symptoms and Dynamic Interactions of Depressive Symptoms in Older Chinese Adults: A Longitudinal Network Analysis

**DOI:** 10.1155/da/8078557

**Published:** 2025-07-23

**Authors:** Yue Feng, Li Chen, Qi Yuan, Lin Ma, Wen Zhao, Lu Bai, Jing Chen

**Affiliations:** ^1^Department of Gynecological Nursing, West China Second University Hospital, Sichuan University, Chengdu, Sichuan, China; ^2^Key Laboratory of Birth Defects and Related Diseases of Women and Children (Sichuan University), Ministry of Education, Chengdu, China; ^3^Department of Operating Room Nursing, West China Second University Hospital, Sichuan University, Chengdu, Sichuan, China; ^4^Department of Pediatric Genetics, Metabolism and Endocrinology Nursing, West China Second University Hospital, Sichuan University, Chengdu, Sichuan, China

**Keywords:** cross-lagged panel network, depression, network analysis, psychopathology

## Abstract

**Background:** Depressive symptoms in older adults are associated with adverse psychosocial outcomes. Understanding how depressive symptoms interrelate can enhance intervention strategies. While network analysis has advanced our comprehension of depressive symptom structure, few studies have explored dynamic interactions in older populations. This study examined both cross-sectional and longitudinal networks of depressive symptoms in older adults to identify core symptoms and symptom interactions over time.

**Methods:** Participants aged 60 and older with complete two-wave data (baseline: 2018; follow-up: 2020) from the China Health and Retirement Longitudinal Study (CHARLS) were included (*N* = 6621). Depressive symptoms were assessed using the 10-item Center for Epidemiologic Studies Depression Scale (CESD-10), administered face-to-face by trained interviewers. Cross-sectional networks were estimated using the Ising model for each time point, and a cross-lagged panel network (CLPN) model was applied to examine longitudinal symptom interactions over time. Network accuracy and stability were assessed through bootstrap procedures.

**Results:** Participants had a mean age of 67.34 years, 52% male, and 93.7% Han ethnicity. “Felt depressed” (*r*_*s*_ = 1.244 at Wave 1, *r*_*s*_ = 1.251 at Wave 2) demonstrated the highest strength centrality in both cross-sectional networks. Node strength exhibited strong stability (correlation stability [CS]-coefficient = 0.75 for both waves). The presence of edges (*φ* = 0.802; *p* < 0.001) and edge weights (*ρ* = 0.921, *p* < 0.001) across two cross-sectional networks showed high reproducibility. In the longitudinal network, “lack of happiness” showed the highest out-expected influence (out-EI; *r* = 1.404), followed by “felt depressed” (*r* = 0.994). Both in-expected influence (in-EI) and out-EI showed acceptable stability (CS-coefficient = 0.594).

**Conclusions:** Targeting core symptoms, such as “felt depressed” and “lack of happiness” may disrupt depressive symptom networks and reduce overall depression severity, informing precision interventions in older adults. Clinicians could prioritize these symptoms in screening and treatment. Future research should explore whether symptom-targeted interventions can reshape network structures over time.

## 1. Introduction

The global challenge of population aging is growing more severe, presenting significant obstacles to preserving and enhancing human health and socioeconomic development, notably in China [1]. China boasts the largest elderly population worldwide, with more than 200 million people aged 60 and above, accounting for 18.70% of the total population [2]. As the population ages, there has been widespread public concern directed toward the primary ailments afflicting older adults, including chronic disease, disabilities, and mental disorders [3]. Studies have reported that 30.6% of the Chinese older adults aged 65 receiving primary care had depressive symptoms [4], and 20.3% had a depressive disorder [5]. The prevalence rate of major depressive disorder (MDD) in Chinese aged 55 years was higher than in 18–54 years [6]. This prevalence rate has been increasing, and the trend is expected to persist in the upcoming decades, contributing to a growing disease burden [7]. Compared to depression in younger age groups, older adults with depression are more likely to have multiple concurrent medical disorders and cognitive impairments [8], along with a higher rate of relapse and less efficacious antidepressants [9]. Depressive symptoms in older adults are associated with increased disability, reduced productivity, impaired interpersonal relationships, diminished overall health, and elevated risk of mortality [10]. So, there is a significant demand for effective interventions for this disadvantaged group.

Depression has been extensively researched from social sciences to genetics over the last century [11]. Most previous studies have relied on summative scoring, which is essential in advancing population-level screening. For example, Zhong et al. [4] estimated the prevalence of depressive symptoms among older Chinese adults using a specific cutoff score from a depression screening tool. Their findings highlight the urgent need to integrate mental health services into primary health care. However, a summative score that aggregates depressive symptoms may not accurately reflect the unique symptom patterns experienced by individuals, potentially leading to underdiagnosis or overdiagnosis [12]. For instance, in depression, anhedonia might hold greater diagnostic significance than sleep disturbances for some patients, but summative scores treat them equally [13].

In addition to summative scoring, many researchers have employed latent variable models of depression, aiming to uncover underlying dimensions of factors that explain the co-occurrence of depressive symptoms [14]. However, latent variable models can obscure the distinct relationships between individual symptoms and their varying levels of importance of influence within the overall symptomatology [15, 16]. Given that depression is a heterogeneous condition, symptoms such as “feeling depressed” or “fatigue” may influence each other differently across individuals, which latent variable models do not capture well [12].

To address these limitations, the network theory of psychopathology has emerged as an alternative framework that views mental disorders as systems of causally interacting symptoms rather than the outcome of a single latent factor [17, 18]. In this approach, symptoms are represented as nodes, and the relationships between them are edges, capturing the structure of the disorder at the symptom level. Symptoms can directly reinforce one another in the network model. For example, insomnia can lead to fatigue, low mood, and difficulty making decisions [19]. Within the networks, central nodes, characterized by stronger relationships with other nodes, hold significance in the network structure and contribute significantly to the progression and persistence of mental disorders [20]. Targeting these core symptoms has been proposed as a promising intervention strategy, as alleviating them may lead to widespread improvements across the symptom network [21, 22]. It is essential to determine the intricate relationships among symptoms if we want to develop effective and precise psychotherapeutic interventions for older adults with depressive symptoms. And a better understanding of the core symptoms is necessary.

In recent years, the network approach has been used increasingly to explore the symptom structure of psychopathologies of depression in older people. For example, Zhang et al. [23] found that feeling sadness, uncontrolled worry, and having trouble relaxing were central symptoms in the anxiety-depression network among disabled older adults aged over 65 in China. Kim et al. [13] indicated that central symptoms were hopelessness, emptiness, and worthlessness in the depression network of older adults aged 60 or over. However, these studies on psychopathologic symptom networks of depression were based on cross-sectional data, which cannot assess the directional relationships and temporal sequencing between symptoms. Exploring longitudinal networks can facilitate the analysis of the dynamic symptom-level interrelationships of depressive symptoms across time. Several studies have examined the dynamic network of depressive symptoms. Schlechter et al. [24] conducted cross-lagged panel network (CLPN) models of depressive symptoms among adults aged 50 and above; they found that “everything an effort,” “could not get going,” and “loneliness” were core symptoms. Zhao et al. [19] explored the longitudinal features of depressive symptoms during the COVID-19 pandemic among 860 Chinese college students (mean age = 20.6) using network analysis. They found that fatigue emerged as the most impactful symptom, with its occurrence potentially triggering other depressive symptoms. Zhu et al. [21] found that feeling fearful was the strongest predictor of dynamic depressive symptoms among adults aged 45 or over. While these studies provided good perspectives for further exploration of the interaction between depressive symptoms, they primarily focus on younger or general adult populations. There remains a notable gap in longitudinal network studies focusing on adults aged 60 and above.

In summary, cross-sectional network analysis has been widely used to investigate depressive symptoms across diverse populations [18], including older adults [13, 23]. While longitudinal network studies examining depressive symptoms have started to emerge [24–26], research specifically targeting adults aged 60 and above remains limited. Although cross-sectional networks have provided valuable insights, they fail to capture dynamic relationships between symptoms over time [27]. Therefore, its crucial to examine the longitudinal relationships of depressive symptoms over time in a sufficiently large and representative sample of older adults, especially when the ultimate objective is to use network targets for enhancing clinical intervention. In this study, we aimed to explore the longitudinal network of depressive symptoms across two time points, compare the cross-sectional networks across different time points, and evaluate the consistency of the network over time.

## 2. Methods

### 2.1. Participants and Procedure

Data were obtained from the China Health and Retirement Longitudinal Study (CHARLS). CHARLS is a nationally representative longitudinal survey conducted by Peking University. It focuses on adults aged 45 and older in China, assessing their social, economic, and health status within the community. CHARLS employed a multistage stratified probability proportional to size (PPS) sampling approach, which provides a nationally representative sample for our study. The baseline survey from May 2011 to March 2012 encompassed approximately 10,000 households and 17,500 individuals in 150 counties/districts and 450 villages/resident committees. Four follow-up surveys were conducted in 2013, 2015, 2018, and 2020. Approval for data collection was obtained from the institutional review board at Peking University (IRB00001052-11014). Written informed consent was obtained from everyone. Details about this cohort's objectives, design, methods, and ethical issues can be found elsewhere [28, 29].

This study used the most recent two waves of data from CHARLS in 2018 (Wave 1) and 2020 (Wave 2). Data for both waves were gathered between July and September of each survey year. In our study, participants aged 60 and older were classified as older adults, following the World Health Organization definition, which designates individuals aged 60 and above as “older adults” category [30]. To explore the longitudinal association between depressive symptoms in older adults, we focused on participants who were aged 60 or older at the initial assessment (Wave 1, 2018) and who completed the follow-up in 2020. As network analysis does not accommodate missing data, participants with incomplete social-demographic and Center for Epidemiologic Studies Depression Scale (CESD) data in any of the waves (Wave 1 in 2018 and Wave 2 in 2020) were excluded from our further analysis. Each participant had a unique identifier, allowing us to match data from Wave 1 to Wave 2 to the same individuals. Finally, a total of 6621 participants were included in our study. The detailed inclusion process is shown in Supporting Information 1: Figure S1.

### 2.2. Measures

#### 2.2.1. Social-Demographic Characteristics

Social-demographic characteristics included age (in years), gender, marital status, ethnicity, educational level, current smoking, alcohol consumption (more than once a month vs. less than once a month), whether living with a spouse, physical activity (moderate or vigorous activity no less than once per week vs moderate or vigorous activity less than once per week), chronic diseases (including hypertension, diabetes, stroke, heart diseases, cancer, chronic lung diseases, liver diseases, cardiovascular diseases, kidney disease, stomach or other digestive diseases, psychiatric disease, memory-related disease, arthritis or rheumatism, and asthma), and self-reported health status (range from 1–5, 1 = very poor, 5 = very good).

#### 2.2.2. Depressive Symptoms

Depressive symptoms were measured by the CESD-10 in CHARLS. The original CES-D consisted of 20 items, which were later adapted into a 10-item shorter version for older adults by Anderson to remove highly redundant items and reduce the respondent burden [31]. Despite being developed over two decades, the CESD-10 has consistently demonstrated adequate reliability and validity in detecting depressive symptoms among the older population in China [32, 33].

In CHARLS, respondents were asked to rate how often they felt each of the 10 depressive symptoms during the past week. The 10 depressive symptoms included in CESD-10 were being bothered by things, having trouble keeping in mind, feeling depressed, feeling everything you did was an effort, feeling hopeful, feeling fearful, being sleepless, feeling happy, feeling lonely, and inability to get going. Each item was scored on a four-point Likert scale: 0 (rarely), 1 (some days; 1–2 days per week), 2 (occasionally; 3–4 days per week), and 3 (most of the time; 5–7 days per week) [34]. Due to many participants in both waves providing “rarely” as their responses, a binary coding approach was applied to CESD-10 to conduct network analysis to avoid zero cell counts for different response categories when estimating polychoric correlations (0 = rarely, 1 = at least sometimes) [35, 36]. The two positive-worded items (feeling hopeful and feeling happy) were first reverse-coded and then binary-coded (hopelessness and lack of happiness) [37]. The Cronbach's alpha of the 10-item CES-D in our sample was 0.816 in Wave 1 and 0.799 in Wave 2.

### 2.3. Data Analysis

Descriptive statistics of continuous variables were reported using means with standard deviations (SDs). Categorical variables were presented as frequencies with percentages. We used McNemar's test to compare the frequencies of each depressive symptom between two waves. All these statistical tests conducted using SPSS 29.0 were two-tailed, with significance levels set at both 0.05 and 0.001 where applicable.

The cross-sectional and CLPN analysis of depressive symptoms was performed using R software 4.3.1 and R Studio 2023.06.1 + 524.

#### 2.3.1. The Cross-Sectional Network Analysis

We constructed separate cross-sectional networks of depressive symptoms in Waves 1 and 2. We conducted network estimation, centrality estimation, network stability, and accuracy assessment for each network. Then, the two networks were compared (Wave 1 vs. Wave 2). The network estimation and visualization were conducted using R-packages qgraph [38] and bootnet [39]. The network consisted of 10 nodes, each representing a depressive symptom, with 45 edge weights estimated to capture the pairwise relationships between them. Each node represented one psychological symptom of depression. The edge represented the partial correlation coefficients between two nodes after conditioning on all other variables in the dataset [39]. Because the variables measuring the depressive symptoms are binary, the Ising model estimation method was used [40]. This technique is based on node-wise binary logistic regression, employing the Least Absolute Shrinkage and Selection Operator (LASSO) regularization procedure and the Extended Bayesian Information Criterion (EBIC) for model selection [41]. The EBIC hyperparameter's value was 0.5 to obtain a reasonable trade-off between sparsity and model fit, preventing overfitting while still capturing essential relationships between nodes [39, 42–44].

Following methodological recommendations for cross-sectional network analysis [45], we focused on strength centrality as the primary metric due to its reliability in psychopathology networks. The strength centrality reflects the magnitude of the relationship between a node and all other nodes in the network [46]. While closeness and betweenness centrality were calculated for completeness, these metrics were reported in Supporting Information 1 for transparency and exploratory purposes only, as their instability in cross-sectional designs limits interpretability [45].

Network accuracy and stability were assessed using R-packages bootnet [39]. Nonparametric bootstrapping was applied to estimate edge weights and their accuracy at 95% confidence intervals (CIs) by sampling the data 1000 times, with narrower CIs representing a more precise network. We used bootstrapping to assess differences in centrality estimates and edge weights. We checked the stability of the centrality indices by calculating correlation stability (CS) coefficients using case-dropping bootstrapping [41, 46]. A CS-coefficient value should be no less than 0.25 and preferably above 0.5 [39].

We employed a combination of statistical methods to evaluate the consistency and replicability of cross-sectional networks between Waves 1 and 2. Specifically, phi correlation (*φ*) assessed the association between edge presence and absence, and Spearman rank correlation analyzed the relationship between edge weights and node-based centrality. Invariance testing checked for maximum edge weight differences. Additionally, we explored global strength disparities between the two networks. A previous study by Horvath [41] used the same approach to assess the replicability of network relationships. These were conducted using R-packages Psych [47] and NetworkComparisonTest [40].

#### 2.3.2. The CLPN Analysis

The longitudinal network of depressive symptoms from Wave 1 to Wave 2 was generated using the CLPN model [48]. CLPNs operationalize dynamic symptom interactions through 10 separate binary logistic regression models with LASSO regularization. For each depressive symptom at Wave 2, two types of effects were specified: the autoregressive effects (prediction of a symptom at Wave 2 by its own level at Wave 1) and cross-lagged effects (prediction of one symptom at Wave 2 by a different symptom at Wave 1). LASSO introduces a penalty term (lambda, *λ*) that shrinks some regression coefficients to exactly zero, effectively performing variable selection. The predictors in all models are depressive symptoms in Wave 1, and the outcomes are depressive symptoms in Wave 2. Ten-fold cross-validation was used to estimate the optimal *λ* values for regularization, the deviance statistic guiding the selection. The one-standard-error rule was applied to balance model parsimony and fit. We identified the *λ* value (*λ*_min) that achieved the minimum cross-validated deviance and selected the largest *λ* value within one standard error of the minimum (*λ*_1SE) [49]. Cross-lagged binary logistic LASSO regression was estimated using R-packages glmnet [49].

Standardized out-expected influence (out-EI) and in-expected influence (in-EI) centrality estimates were calculated to assess the importance of nodes in the longitudinal network. Out-EI measures the degree to which a symptom at Wave 1 predicts other symptoms at Wave 2, whereas the in-EI quantifies the degree to which a symptom at Wave 2 is predicted by others at Wave 1 [50]. The high out-EI symptoms can potentially target symptoms in longitudinal studies, and intervention may lead to the relief of other symptoms [51].

The methods of assessing the stability and accuracy of the cross-lagged network were the same as those of the cross-sectional network.

#### 2.3.3. Comparative Analysis

To assess the robustness of our main findings derived from binary CESD-10 responses, we conducted a supporting analysis using the original 4-point Likert-scale data (from 0 to 3). Gaussian graphical models (GGMs) were estimated to construct cross-sectional networks. A CLPN was also constructed to model temporal associations between symptoms from Wave 1 to Wave 2. All other analytical procedures, including centrality estimation, bootstrapping for accuracy, and network comparison tests, were identical to the main analysis.

## 3. Results

### 3.1. Participants' Characteristics and Symptoms Descriptive Statistics

Table 1 presents the participants' characteristics (*N* = 6621). The mean age was 67.64 ± 3.00 years, ranging from 60 to 91 years. Most of our participants were Han Chinese (93.7%), married (83.6%), had obtained a middle school diploma or less (64.9%), and lived with a spouse (80.2%).

Table 2 presents the proportion of individuals who responded with a value of “1” (indicating at least some level of the symptom) for each depressive symptom in Waves 1 and 2, along with the results of McNemar's test evaluating changes over time. “D5: hopelessness” was the most prevalent symptom in both waves (61.86% in Wave 1 and 67.04% in Wave 2). A significant but slight increase was shown in “D1: bothered by things” (*c*^2^ = 21.926, *p* < 0.001), “D2: had trouble keeping in mind” (*c*^2^ = 21.935, *p* < 0.001), “D5: hopelessness” (*c*^2^ = 49.373, *p* < 0.001), “D6: felt fear” (*c*^2^ = 5.385, *p* < 0.05), “D8: lack of happiness” (*c*^2^ = 42.407, *p* < 0.001), and “D10: could not get going” (*c*^2^ = 8.862, *p* < 0.05) from Wave 1 to Wave 2, as determined by McNemar's test.

### 3.2. Cross-Sectional Network

**Figure 1:**
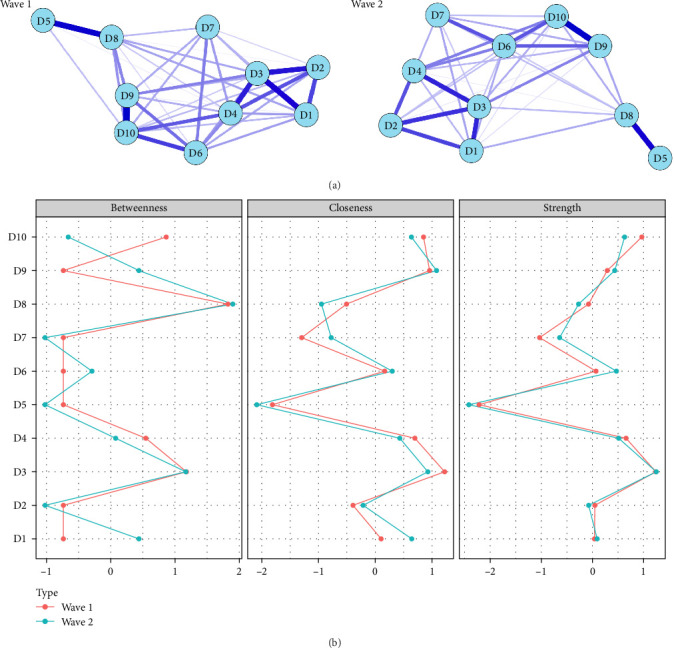
Cross-sectional networks (a) and centrality indices (b) of depressive symptoms in Wave 1 and Wave 2. The thickness of the edges represents the magnitude of the correlation. Blue edges = positive correlations. Node abbreviations: D1: bothered by things; D2: had trouble keeping in mind; D3: felt depressed; D4: everything an effort; D5: hopelessness, D6: Felt fear; D7: sleep was restless; D8: lack of happiness; D9: felt lonely; D10: could not get going.

Cross-sectional network visualization of depressive symptoms in Waves 1 and 2 was presented in Figure 1a. The varying thickness of the edges represents the magnitude of the correlation between symptoms, with blue for positive correlations and red for negative. We observed the strongest connections within the paired symptoms in each network. For example, strong connections were observed between “D9: felt lonely” and “D10: could not get going”, “D5: hopelessness” and “D8: lack of happiness”, “D1: bothered by things” and “D3: felt depressed”, both in Waves 1 and 2. Supporting Information 1: Table S1 presents the edge weights of the cross-sectional networks. Thirty-nine edges (86.7%) were nonzero edges with a mean weight of 0.480 in Wave 1, and 36 edges (80.0%) were nonzero edges with a mean weight of 0.462 in Wave 2. The most substantial positive relationship was observed between “D5: hopelessness” and “D8: lack of happiness” in Wave 1 (edge weight = 1.48) and between “D9: felt lonely” and “D10: could not get going” in Wave 2 (edge weight = 1.59).

Standardized centrality indices of both networks were presented in Figure 1b and Supporting Information 1: Table S2. According to the results of centrality indices, the top three symptoms in terms of strength in Wave 1 are as follows: “felt depressed” (*r*_*s*_ = 1.244), “could not get going” (*r*_*s*_ = 0.968), and “everything an effort” (*r*_*s*_ = 0.659). The top three symptoms in terms of strength in Wave 2 are as follows: “felt depressed” (*r*_*s*_ = 1.251), “could not get going” (*r*_*s*_ = 0.633), and “everything an effort” (*r*_*s*_ = 0.514). “Felt depressed” was the most central symptom in both Waves 1 and 2, followed by “could not get going” and “everything an effort”.

**Figure 2:**
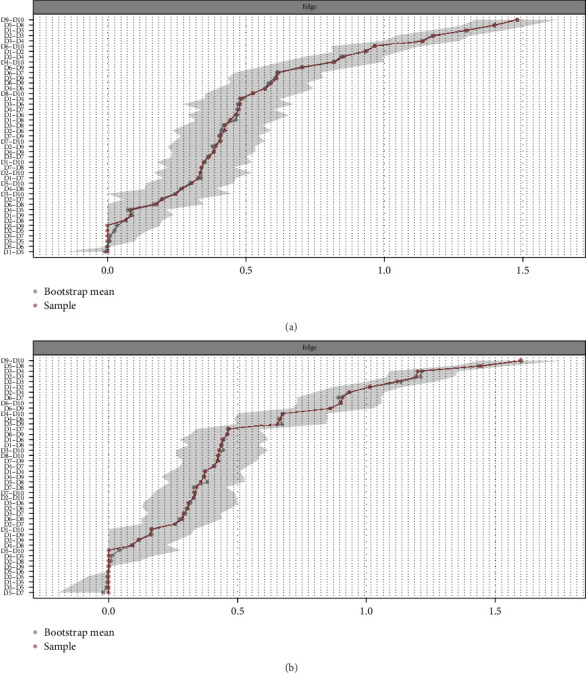
Accuracy of edge weights of cross-sectional networks of depressive symptoms in Wave 1 (a) and Wave 2 (b). The red lines indicate edge weights from our samples. Black lines are edge weights generated based on 1000 random bootstrap samples. Consistency between red lines and black lines suggests high accuracy. Grey areas are bootstrap confidence intervals (CIs), with narrower intervals indicating higher precision. If the red line falls within the grey CI, the edge is considered stable. The *X*-axis represents the effect size value between two nodes. The *y*-axis represents the combination of two nodes in the cross-sectional network.

**Figure 3:**
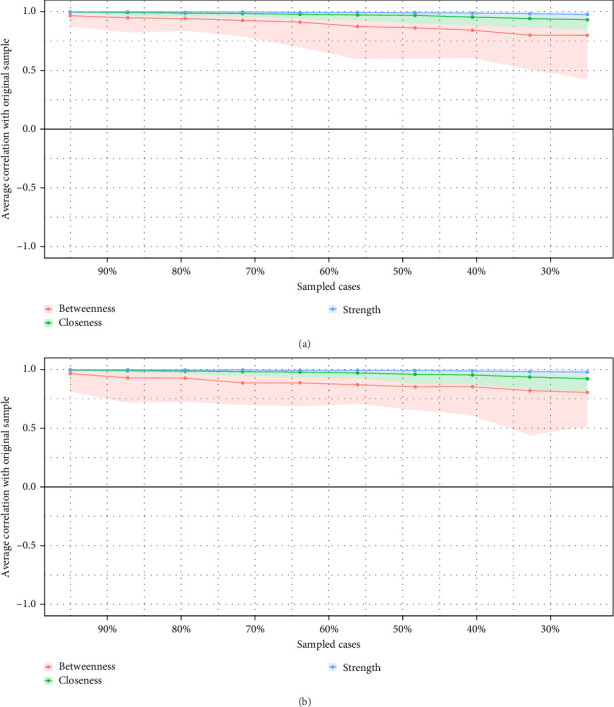
Stability of centrality indices for cross-sectional networks of depressive symptoms in Wave 1 (a) and Wave 2 (b). Lines link the means of correlations from subsets with an increasing number of excluded participants. Colored areas indicate the range of correlations from the 2.5th to the 97.5th quantile.

The accuracy analysis of the cross-sectional networks in Waves 1 and 2 is presented in Figure 2, which shows the high accuracy of the networks.  Figure 3 shows the case-drop bootstrapping results. Node strength (CS-coefficient = 0.75 for both waves) exhibited strong stability in Waves 1 and 2.

The presence of edges (*φ* = 0.802; *p* < 0.001) and the edge weights (*ρ* = 0.921; *p* < 0.001) across cross-sectional networks of Waves 1 and 2 showed high levels of stability or reproducibility. Network invariance tests indicated nonsignificant maximum differences in any of the edge weights (*M* = 0.305; *p*=0.297) between the two cross-sectional networks. A significant decrease in global strength was observed from Wave 1(*S* = 21.610) to Wave 2 (*S* = 20.775) (Δ*S* = 0.835; *p*=0.020).

### 3.3. CLPN

**Figure 4:**
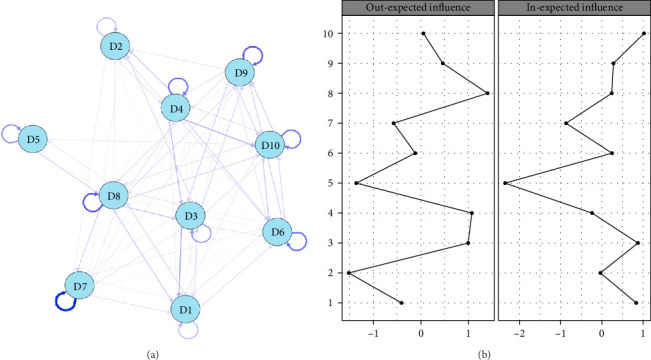
The longitudinal network (a) and standardized centrality indices (b) of depressive symptoms between Wave 1 and Wave 2. The thickness of the edges represents the magnitude of the correlation. Blue edges = positive correlations. The arrows on the lines show the predictive effects of depressive symptoms in Wave 1 on symptoms in Wave 2. Loop arrows represent autoregressive effects. Node abbreviations: D1: bothered by things; D2: had trouble keeping in mind; D3: felt depressed; D4: everything an effort; D5: hopelessness; D6: felt fear; D7: sleep was restless; D8: lack of happiness; D9: felt lonely; D10: could not get going.

The CLPN of depressive symptoms from Waves 1 to 2 was presented in Figure 4a. All edge weights were presented in Supporting Information 1: Table S3. In total, 76 edges (76.0%) were nonzero with a mean weight of 0.157. Symptoms with the greatest autoregression coefficients were “D7: sleep was restless” (*β* = 1.16), followed by “D8: lack of happiness” (*β* = 0.75). Figure 4b presents the standardized centrality indices of the dynamic network. Supporting Information 1: Table S4 and Supporting Information 1: Figure S2 present the centrality indices and the accuracy indicators. In the longitudinal symptom network, “D8: lack of happiness” at Wave 1 showed the highest value of out-EI (*r* = 1.404), significantly predicting “D1: bothered by things” (*β* = 0.26), “D3: felt depressed” (*β* = 0.26), “D7: sleep was restless” (*β* = 0.22), “D10: could not get going” (*β* = 0.20), and “D9: felt lonely” (*β* = 0.17) at Wave 2. Similarly, “D3: felt depressed” had the second highest out-EI (*r* = 0.994) at Wave 1, predicting “D1: bothered by things” (*β* = 0.39), “D9: felt lonely” (*β* = 0.21), “D2: had trouble keeping in mind” (*β* = 0.19), “D10: could not get going” (*β* = 0.15) at Wave 2. “Could not get going,” “felt depressed,” and “bothered by things” had the strongest in-EI. The CS-coefficients of both in-EI and out-EI were 0.594, which showed moderate stability (values >0.5 are considered acceptable).

### 3.4. Comparative Analysis

The results of the cross-sectional and CLPN estimation using original Likert-scale data were presented in Supporting Information 2: Appendix file. The comparative results demonstrated consistency in centrality rankings and network structures across the two approaches.

## 4. Discussion

This study examined the cross-sectional and longitudinal networks of depressive symptoms in a large sample of older adults and evaluated their replicability and stability. Regarding changes during the follow-up, a significant but slight increase in the proportion was shown in several symptoms. At the same time, the network structure remained relatively stable and did not experience significant change over this period. Overall, the findings indicate varying strengths in symptom associations, highlighting that individual depressive symptoms do not carry equal importance in the network. This diversity underscores the significance of investigating connections between individual symptoms rather than relying on composite sum scores or diagnoses [52].

In examining cross-sectional networks, we identified that feeling depressed was the most central depressive symptom at both time points among older Chinese adults. This symptom had the highest strength centrality values in both Waves 1 and 2, indicating that it was the most strongly associated with other depressive symptoms in the network. This finding is consistent with previous studies [53] that identified a depressed mood as the core symptom of depression in older people. A depressed mood is commonly recognized as the primary symptom of depression [54]. Current depression screening tools do not hierarchically prioritize symptoms across emotional, cognitive, and neurological domains, with only fundamental criteria like depressed mood and anhedonia universally emphasized [55]. Our network results provided empirical support for the centrality of depressed mood. Specifically, the stability of this symptom's centrality in the depressive network from 2018 to 2020 resonated with both the depression theoretical framework [55] and contemporary network-based investigations [20].

Another study using the Patient Health Questionnaire-9 (PHQ-9) analyzed depressive symptom networks and found that anhedonia and feeling depressed were the core symptoms in older people with subjective memory complaints [53]. It is essential to note that the CESD-10 and PHQ-9 assess depressive symptoms using distinct item sets, though they share some common symptoms, such as anhedonia, depressed mood, sleep problems, concentration difficulties, and low energy [53]. These differences in item content may shape the symptom network differently, potentially leading to variations in which symptoms are identified as most central. Despite these differences, the identification of a depressed mood as a core symptom across studies using various scales suggests a degree of consistency. Several researchers have proposed that addressing the symptoms identified as central in cross-sectional networks may lead to significant treatment advancements [56, 57]. This implies that cross-sectional network analysis could potentially identify valuable targets for treatment. However, since cross-sectional networks can't reveal the causal relationships between symptoms, researchers have called for caution in interpreting core symptoms in cross-sectional networks and for more longitudinal network studies to uncover causal relationships [58].

The edge weight analysis in the cross-sectional networks identifies the strongest pairwise association between feeling lonely, could not get going and hopelessness, lack of happiness. Our findings align with Zhang et al. [37] and are partly consistent with Ma et al. [59], who reported similar depressive symptom patterns in older Chinese adults. These associations suggest that interventions addressing one symptom in each pair may alleviate the other symptom simultaneously [60]. Previous research supported this idea [61], where interventions aimed at increasing happiness among older adults residing in nursing homes helped reduce feelings of hopelessness and depression in long-term care facilities. The results highlighted the potential for interventions that address interconnected symptoms simultaneously rather than treating symptoms in isolation.

Our longitudinal network revealed that the symptom with the greatest autoregressive effects was sleep restlessnes*s*, suggesting that the sleep problems are likely to persist across time points. The strong autoregressive effect of sleep restlessness may reflect its association with chronic health issues, such as persistent medical comorbidities and cognitive impairment, which are commonly prevalent in older adults with depressive symptoms [8]. Moreover, sleep problems are frequently reported as adverse effects of antidepressant medications [8]. Such factors may contribute to persisting and reinforcing sleep problems over time. This finding addressed the importance of targeting sleep restlessness early in interventions to prevent the prolonged continuation of depressive states.

In our study, lack of happiness and feeling depressed showed the highest out-EI values in older adults, indicating that these symptoms played central roles in activating and predicting the emergence of other depressive symptoms. The results were somewhat expected, as anhedonia and depressed mood were recognized as fundamental symptoms of depression [55]. Consistent with our results, a previous longitudinal study in older adults also identified “lack of happiness” as one of the symptoms with the highest out-EI in the depression network [27]. In older adults, persistently low happiness levels can be caused by limited engagement in meaningful or pleasurable activities, social isolation, chronic health conditions, or psychosocial losses such as bereavement [62, 63]. These factors may initiate a self-reinforcing cycle in older adults. Individuals with high anticipatory anhedonia may expect little reward from typically pleasurable activities, reducing their motivation and effort to engage in them. This avoidance further exacerbates anhedonia and reinforces depressive symptoms [64]. Lack of happiness has been considered a primary therapeutic target in MDD. Horne et al. [65] summarized that interventions addressing anhedonia may enhance the efficacy of current antidepressants and inform the development of future therapies. Moreover, positive psychology interventions tailored to older adults (e.g., gratitude exercises and meaningful activity scheduling) have been shown to elevate happiness levels and reduce depressive symptoms [66]. These findings suggest that interventions targeting “lack of happiness” may serve both as a warning sign and a modifiable target for preventive interventions in late-life depression.

The higher out-EI of feeling depressed relative to other symptoms in our depressive symptom network aligns with findings from studies conducted in adolescent populations [67, 68], indicating that a depressed mood can lead to the emergence of additional symptoms over time. Given the centrality of the depressed mood in the network, targeted interventions aimed at alleviating this symptom may be particularly beneficial in preventing the escalation of depressive symptoms. For example, smartphone apps that target depressive mood through subliminal positive word stimulation promise immediate symptom relief in subthreshold depression cases [69]. A recent study found that acute-phase cognitive therapy could reduce depressed mood and low general interest in MDD [70]. However, it is important to note that while network structures and centrality metrics offer valuable insights, we must be cautious in interpreting their implications. Currently, there is a limited examination of whether targeting core symptoms in a network actually reduces overall network connectivity, even though this should theoretically be the case. Future studies could investigate this theoretical hypothesis by targeting core symptoms in the network and capturing changes in the symptom networks during the intervention.

While our study provided valuable insights into the network of depressive symptoms in older adults, several limitations should be acknowledged. Our study created a longitudinal network using only the two most recent and comprehensive waves of data available in the CHARLS database. However, using two-wave data limits the ability to assess the stability of relationships over time. While previous studies indicate that two-wave cross-lagged models can provide valuable insights [71–73], future studies should collect additional data waves to validate and extend these findings. Secondly, as noted in the literature [74], a prerequisite for cross-lagged effect analysis is excluding all potential sources of spuriousness. Our study, however, does not account for certain important covariates, which limits the ability to draw causal conclusions from the observed associations. Therefore, our findings should be interpreted as exploratory and indicative of associations rather than causal relationships. Furthermore, the CESD-10 included fewer items than the original CES-D, which may reduce its ability to capture the full spectrum of depressive symptoms in older adults. Differences in the symptom nodes in the network may lead to different symptom networks. So, this reduction in symptom coverage may result in omitting central symptoms that are particularly relevant to this population, potentially influencing the identified symptom networks and their clinical interpretation. Fried's [11] comparison of seven common depression scales found that depression may encompass a total of 52 symptoms, suggesting that other unmeasured symptoms could play an important role in the depressive symptom network. Additionally, using different scales to screen for depressive symptoms may result in similarities and differences between the constructed symptom networks. By comparing the core symptoms of each scale and the connections between symptoms, we can better understand the overall network of depressive symptoms.

Depressive symptoms were assessed using binary responses, allowing analyses to document the presence or absence of symptoms. However, information regarding the severity of symptoms among those affected could not be obtained. To address potential concerns regarding the loss of information through binary responses of CESD-10, we conducted an additional analysis using the original Likert-scale data. This allowed us to compare the network structure and symptom dynamics estimated from both binary and continuous inputs. The findings revealed consistency across the two modeling approaches. “Felt depressed” consistently emerged as a central symptom across cross-sectional and longitudinal networks. Stability metrics and bootstrapping analyses further supported the robustness of these estimates. Notably, although mean edge weights were lower in the Likert-based models due to the scale characteristics, the relative strengths and predictive relationship between symptoms remained largely unchanged, reinforcing the validity of our main conclusions.

Importantly, depression in clinical settings rarely occurs in isolation, highlighting the need to consider transdiagnostic symptoms that may bridge comorbid conditions [75]. Kaiser et al. [22] suggest that psychomotor symptoms, such as restlessness, can be bridge symptoms, connecting symptoms of depression and anxiety. Although our study did not explore this directly, it provides a useful framework for future research investigating how these symptoms might function in comorbid populations. Future studies should aim to include comorbid populations to examine whether bridge symptoms can be identified within networks of multiple disorders and how these symptoms influence treatment outcomes. Longitudinal analyses could also provide a more dynamic understanding of symptom overlap and its role in network interventions that target shared symptom domains.

## 5. Conclusion

Our study used a nationally representative sample of older adults aged 60 and over in China. Two cross-sectional networks of depressive symptoms were compared using two waves of data from 2018 to 2020, and a longitudinal network was conducted then. Based on node centrality, the most central symptom of the two cross-sectional networks of depressive symptoms was depressed mood. The strongest predictors of depressive symptoms among older adults were the symptoms of depressed mood and lack of happiness. Our study provides valuable information for designing more precise and effective psychotherapeutic interventions for older adults in China, as deactivating the central symptom may also decrease other connected nodes. Our findings extend the understanding of depressive symptoms in older adults and suggest that depressed mood and lack of happiness may be promising targets for intervention.

## Figures and Tables

**Table 1:** Social-demographic information at enrollment (*N* = 6621).

Variables	Mean ± SD (*n*, %)
Age, mean (SD), year	67.64 ± 3.00
Gender, male	3441 (52.0%)
Marital status	
Married/cohabitated	5535 (83.6%)
Widowed/divorced/separated	1050 (15.9%)
Never married	36 (0.5%)
Ethnicity	
Han	6202 (93.7%)
Minority	419 (6.3%)
Education attainment	
Illiterate	1594 (24.1%)
Middle school and below	4296 (64.9%)
High school and upper	731 (11.0%)
Current smoking	260 (3.9%)
Alcohol consumption	1812 (27.4%)
Living with spouse	5307 (80.2%)
Physical activity	1931 (29.2%)
Chronic diseases	
Hypertension	756 (11.4%)
Diabetes	382 (5.8%)
Dyslipidemia	666 (10.1%)
Cancer	76 (1.1%)
Chronic lung diseases	351 (5.3%)
Liver diseases	234 (3.5%)
Heart diseases	511 (7.7%)
Stroke	399 (6.0%)
Kidney diseases	277 (4.2%)
Stomach or other digestive diseases	466 (7.0%)
Psychiatric diseases	61 (0.9%)
Memory-related diseases	148 (2.2%
Arthritis or rheumatism	451 (6.8%)
Asthma	159 (2.4%)
Self-rated health, mean (SD)	3.00 ± 1.00

**Table 2:** Changes in symptom proportion across two waves of measurement (*N* = 6621).

Node	Item	Wave 1	Wave 2	*χ* ^2^
D1	Bothered by things	3007 (45.42%)	3241 (48.95%)	21.926*⁣*^*∗∗*^
D2	Had trouble keeping in mind	3096 (46.76%)	3364 (50.81%)	21.935*⁣*^*∗∗*^
D3	Felt depressed	3140 (47.42%)	3152 (47.61%)	0.052
D4	Everything an effort	3203 (48.38%)	3274 (49.45%)	2.091
D5	Hopelessness	4096 (61.86%)	4439 (67.04%)	49.373*⁣*^*∗∗*^
D6	Felt fear	1348 (20.36%)	1442 (21.78%)	5.385*⁣*^*∗*^
D7	Sleep was restless	3471 (52.42%)	3386 (51.14%)	3.333
D8	Lack of happiness	3294 (49.75%)	3609 (54.51%)	42.407*⁣*^*∗∗*^
D9	Felt lonely	2007 (30.31%)	2006 (30.30%)	0.000
D10	Could not get going	1521 (22.97%)	1646 (24.86%)	8.862*⁣*^*∗*^

*⁣* ^*∗*^ *p* < 0.05.

*⁣* ^*∗∗*^ *p* < 0.001.

## Data Availability

Publicly available datasets were analyzed in this study. This data can be found here: http://charls.pku.edu.cn. R script and simulated example data are publicly available at: https://osf.io/hkcxv/.

## Supporting Information

Supporting Information 1 The following supporting information is available with this article: The supporting file contains supporting tables and figures that provide extended data and visualizations supporting the results discussed in the main text.

Supporting Information 2 The appendix file presents an additional methodological analysis. It details the results of network analyses performed using continuous variables, serving as a comparative validation to the primary analysis using binary variables presented in the main manuscript. This comparison underscores the reliability of the methodological approach employed.
