# Supplementary material for: Core Symptoms and Dynamic Interactions of Depressive Symptoms in Older Chinese Adults: A Longitudinal Network Analysis
Source: Depress Anxiety. 2025 Jul 23;2025:8078557. doi: 10.1155/da/8078557 (PMC12310319; doi:10.1155/da/8078557)
Supplement: Supporting Information 1 — The following supporting information is available with this article: The supporting file contains supporting tables and figures that provide extended data and visualizations supporting the results discussed in the main text. [file 8078557.f1.docx]

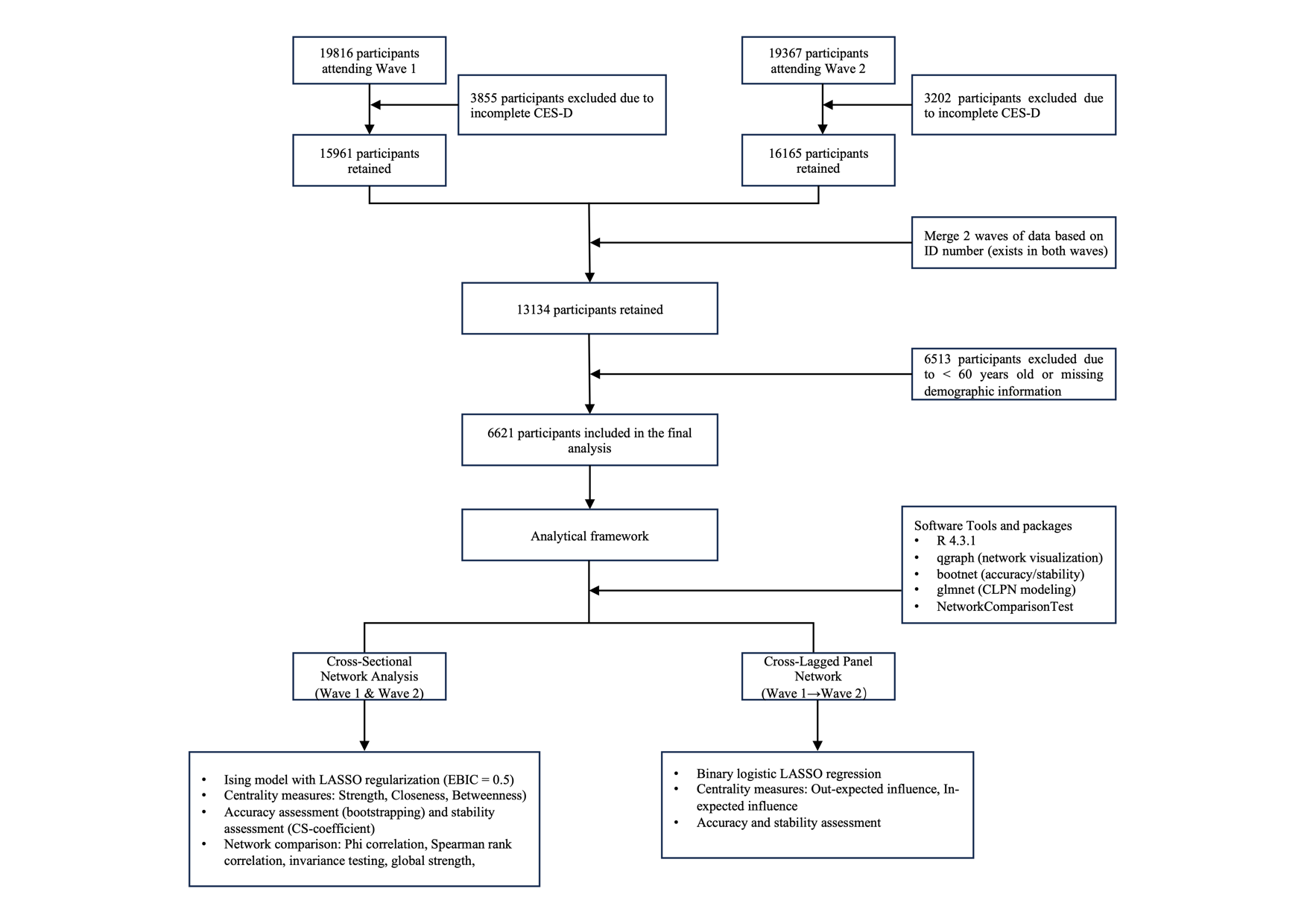


**Supplementary Figure 1.** Participants selection and analytical framework diagram

**Supplementary Table 1.** Edge weights of the cross-sectional networks of depressive symptoms in Wave 1 and Wave 2

|  | **D1** | **D2** | **D3** | **D4** | **D5** | **D6** | **D7** | **D8** | **D9** | **D10** |
| --- | --- | --- | --- | --- | --- | --- | --- | --- | --- | --- |
| **D1** | - | 0.93 | 1.29 | 0.48 | 0.00 | 0.46 | 0.33 | 0.44 | 0.09 | 0.35 |
| **D2** | 1.01 | - | 1.18 | 0.85 | 0.00 | 0.42 | 0.20 | 0.06 | 0.39 | 0.34 |
| **D3** | 1.19 | 1.12 | - | 1.14 | 0.00 | 0.48 | 0.37 | 0.42 | 0.61 | 0.25 |
| **D4** | 0.38 | 0.93 | 1.20 | - | 0.09 | 0.57 | 0.47 | 0.27 | 0.39 | 0.82 |
| **D5** | 0.00 | 0.00 | 0.00 | 0.00 | - | 0.00 | 0.00 | 1.40 | 0.00 | 0.31 |
| **D6** | 0.44 | 0.31 | 0.31 | 0.67 | 0.00 | - | 0.62 | 0.18 | 0.71 | 0.97 |
| **D7** | 0.47 | 0.26 | 0.29 | 0.41 | 0.00 | 0.91 | - | 0.34 | 0.41 | 0.41 |
| **D8** | 0.44 | 0.00 | 0.36 | 0.10 | 1.45 | 0.29 | 0.34 | - | 0.59 | 0.52 |
| **D9** | 0.16 | 0.11 | 0.66 | 0.37 | 0.00 | 0.86 | 0.42 | 0.46 | - | 1.48 |
| **D10** | 0.17 | 0.33 | 0.43 | 0.68 | 0.00 | 0.90 | 0.34` | 0.43 | 1.59 | - |

Note: Edge weights of the cross-sectional network in Wave 1 are shown above the diagonal, while the edge weights of the network in Wave 2 are displayed below the diagonal.

D1: Bothered by things, D2: Had trouble keeping in mind, D3: Felt depressed, D4: Everything an effort, D5: Hopelessness, D6: Felt fear, D7: Sleep was restless, D8: Lack of happiness, D9: Felt lonely, D10: Could not get going.

**Supplementary Table 2.** Centrality indices in Wave 1 and Wave 2

|  | **Wave 1** | | |  | **Wave 2** | | |
| --- | --- | --- | --- | --- | --- | --- | --- |
|  | **Betweenness** | **Closeness** | **Strength** |  | **Betweenness** | **Closeness** | **Strength** |
| **D1** | -0.735 | 0.106 | 0.039 |  | 0.438 | 0.642 | 0.092 |
| **D2** | -0.735 | -0.395 | 0.047 |  | -1.022 | -0.213 | -0.071 |
| **D3** | 1.183 | 1.216 | 1.244 |  | 1.168 | 0.927 | 1.251 |
| **D4** | 0.543 | 0.700 | 0.659 |  | 0.073 | 0.430 | 0.514 |
| **D5** | -0.735 | -1.806 | -2.216 |  | -1.022 | -2.083 | -2.420 |
| **D6** | -0.735 | 0.169 | 0.073 |  | -0.292 | 0.301 | 0.474 |
| **D7** | -0.735 | -1.294 | -1.034 |  | -1.022 | -0.776 | -0.642 |
| **D8** | 1.822 | -0.505 | -0.075 |  | 1.897 | -0.947 | -0.269 |
| **D9** | -0.735 | 0.955 | 0.294 |  | 0.438 | 1.079 | 0.438 |
| **D10** | 0.863 | 0.852 | 0.968 |  | -0.657 | 0.638 | 0.633 |

D1: Bothered by things, D2: Had trouble keeping in mind, D3: Felt depressed, D4: Everything an effort, D5: Hopelessness, D6: Felt fear, D7: Sleep was restless, D8: Lack of happiness, D9: Felt lonely, D10: Could not get going.

**Supplementary Table 3.** Edge weights of a dynamic network of depressive symptoms, from Wave 1 (column) to Wave 2 (row)

|  | **D1** | **D2** | **D3** | **D4** | **D5** | **D6** | **D7** | **D8** | **D9** | **D10** |
| --- | --- | --- | --- | --- | --- | --- | --- | --- | --- | --- |
| **D1** | 0.36 | 0.02 | 0.20 | 0.08 | 0.00 | 0.17 | 0.07 | 0.19 | 0.01 | 0.02 |
| **D2** | 0.00 | 0.34 | 0.04 | 0.14 | 0.00 | 0.00 | 0.09 | 0.00 | 0.00 | 0.00 |
| **D3** | 0.39 | 0.19 | 0.49 | 0.08 | 0.00 | 0.14 | 0.08 | 0.14 | 0.21 | 0.15 |
| **D4** | 0.10 | 0.27 | 0.31 | 0.64 | 0.00 | 0.22 | 0.04 | 0.05 | 0.07 | 0.35 |
| **D5** | 0.00 | 0.00 | 0.00 | 0.00 | 0.54 | 0.00 | 0.00 | 0.22 | 0.00 | 0.12 |
| **D6** | 0.13 | 0.15 | 0.07 | 0.00 | 0.00 | 0.68 | 0.10 | 0.00 | 0.23 | 0.20 |
| **D7** | 0.14 | 0.06 | 0.12 | 0.09 | 0.00 | 0.04 | 1.16 | 0.12 | 0.10 | 0.03 |
| **D8** | 0.26 | 0.11 | 0.26 | 0.15 | 0.12 | 0.06 | 0.22 | 0.75 | 0.17 | 0.20 |
| **D9** | 0.09 | 0.13 | 0.22 | 0.17 | 0.00 | 0.13 | 0.02 | 0.16 | 0.74 | 0.23 |
| **D10** | 0.12 | 0.00 | 0.03 | 0.16 | 0.00 | 0.27 | 0.00 | 0.14 | 0.25 | 0.63 |

D1: Bothered by things, D2: Had trouble keeping in mind, D3: Felt depressed, D4: Everything an effort, D5: Hopelessness, D6: Felt fear, D7: Sleep was restless, D8: Lack of happiness, D9: Felt lonely, D10: Could not get going.

**Supplementary Table 4.** In-Expeted Influence and Out-Expected Influence in the longitudinal network

|  | **In-Expected Influence** | **Out-Expected Influence** |
| --- | --- | --- |
| **D1** | 0.830 | -0.411 |
| **D2** | -0.037 | -1.518 |
| **D3** | 0.873 | 0.994 |
| **D4** | -0.238 | 1.072 |
| **D5** | -2.346 | -1.362 |
| **D6** | 0.243 | -0.120 |
| **D7** | -0.867 | -0.574 |
| **D8** | 0.239 | 1.404 |
| **D9** | 0.282 | 0.462 |
| **D10** | 1.021 | 0.053 |

D1: Bothered by things, D2: Had trouble keeping in mind, D3: Felt depressed, D4: Everything an effort, D5: Hopelessness, D6: Felt fear, D7: Sleep was restless, D8: Lack of happiness, D9: Felt lonely, D10: Could not get going.

**
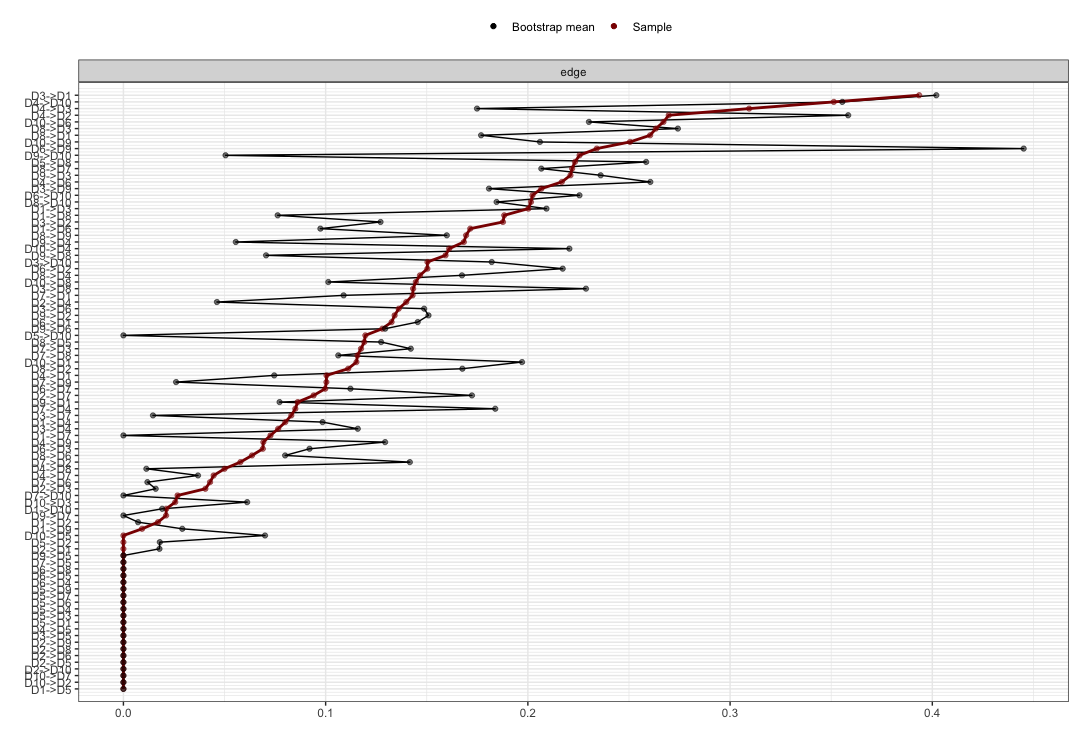
**

**Supplementary Figure 2.** Accuracy of edge weights in the dynamic network of depressive symptoms. Red dots and lines are the edge weights from the sample. Black dots and lines are edge weights from 1000 random bootstrap samples.
