# Supplementary material for: Core Symptoms and Dynamic Interactions of Depressive Symptoms in Older Chinese Adults: A Longitudinal Network Analysis
Source: Depress Anxiety. 2025 Jul 23;2025:8078557. doi: 10.1155/da/8078557 (PMC12310319; doi:10.1155/da/8078557)
Supplement: Supporting Information 2 — The appendix file presents an additional methodological analysis. It details the results of network analyses performed using continuous variables, serving as a comparative validation to the primary analysis using binary variables presented in the main manuscript. This comparison underscores the reliability of the methodological approach employed. [file 8078557.f2.docx]

**Comparative analysis**

In the main analysis, we used the binary responses of the CESD-10 to indicate the presence or absence of each symptom. However, this dichotomization may influence the interpretability and structure of the symptom network. To address this concern and examine the robustness of our findings, we conducted a supplementary analysis using the original 4-point Likert-scale data. Gaussian Graphical Models (GGMs) were estimated to construct cross-sectional networks. A cross-lagged panel network was also constructed to model temporal associations between symptoms from Wave 1 to Wave 2. All other analytical procedures, including centrality estimation, boostrapping for accuracy, and network comparison tests, were identical to the main analysis.

**1. Cross-sectional network**

Cross-sectional network visualization of depressive symptoms in Wave 1 and Wave 2 was depicted in **Appendix Figure 1 (a)**. **Appendix Table 1** presented the edge weights of the cross-sectional networks. 43 edges (95.6%) were non-zero edges with a mean weight of 0.086 in Wave 1, and 40 edges (88.9%) were non-zero edges with a mean weight of 0.083 in Wave 2. The most substantial positive relationship was observed between "D5: hopelessness" and "D8: lack of happiness" in Wave 1 (edge weight = 0.33) and Wave 2 (edge weight = 0.035).

Standardized centrality indices of both networks were presented in **Appendix Figure 1 (b)** and **Appendix Table 2**. According to the results of centrality indices, the top 3 symptoms in terms of strength in Wave 1 are as follows: "felt depressed" (*r_s_* = 1.768), "bothered by things" (*r_s_* = 0.626), and "could not get going" (*r_s_* = 0.487). The top 3 symptoms in terms of strength in Wave 2 are as follows: "felt depressed" (*r_s_* = 1.943), "bothered by things" (*r_s_* = 0.499), and "could not get going" (*r_s_* = 0.383). "Felt depressed" was the most central symptom in both Wave 1 and Wave 2, followed by " Bothered by things" and "Could not get going".

The accuracy analysis of the cross-sectional networks in Wave 1 and Wave 2 was presented in **Appendix Figure 2**. **Appendix Figure 3** showed the case-drop bootstrapping results. Node strength (CS-coefficient = 0.75 for both waves) exhibited strong stability in Wave 1 and Wave 2.

The presence of edges (*φ*=0.759; *p*<0.001) and the edge weights (*ρ*=0.955, *p*<0.001) across cross-sectional networks of Wave 1 and Wave 2 showed high levels of stability or reproducibility. Network invariance tests (*M*=0.056; *p*=0.178) and global strength invariance test (*ΔS*=0.002; *p*=1) indicated non-significant differences between the two cross-sectional networks.

**2. Cross-lagged panel network**

The cross-lagged panel network of depressive symptoms from Wave 1 to Wave 2 was presented in **Appendix Figure 4 (a)**. All edge weights were presented in **Appendix Table 3**. In total, seventy-one edges (71.0%) were non-zero with a mean weight of 0.036. Symptoms with the greatest autoregression coefficients were "D7: sleep was restless" (*β* = 0.35), followed by "D4: everything an effort" and "D9: felt lonely (*β* = 0.20). **Appendix Figure 4 (b)** presented the standardized centrality indices of the dynamic network. **Appendix Table 4** and **Appendix Figure 5** presented the centrality indices and the accuracy indicators. In the longitudinal symptom network, "D4: everything an effort" showed the highest value of out-EI (*r*=1.498), followed by "D3: felt depressed" (*r*=1.272). "Bothered by things" and "felt depressed" had the strongest In-EI. The CS-coefficients of both In-EI and Out-EI were 0.594, which showed moderate stability (values >0.5 are considered acceptable).

**
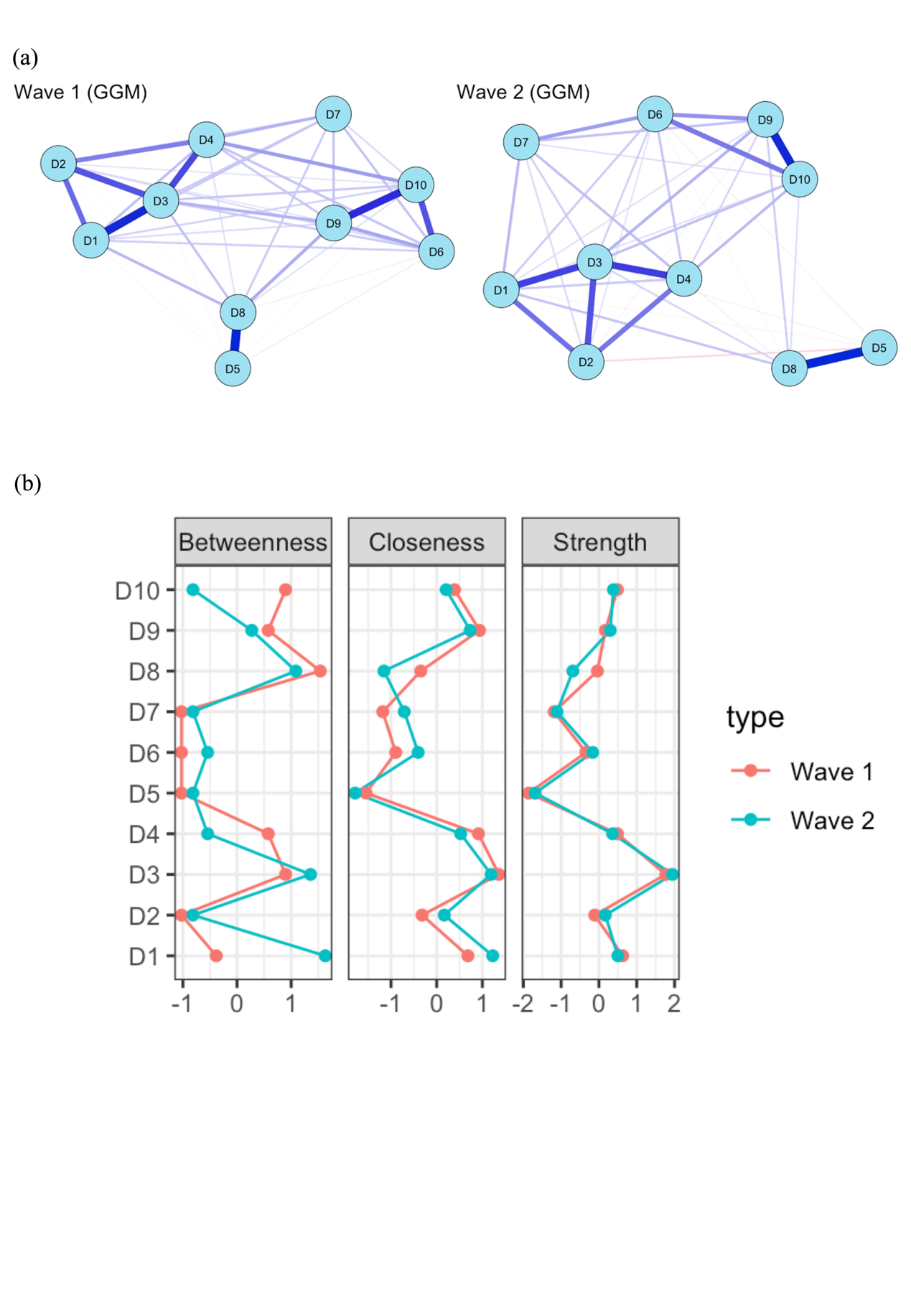
**

**Appendix Figure 1** Cross-sectional networks (a) and centrality indices (b) of depressive symptoms in Wave 1 and Wave 2. The thickness of the edges represents the magnitude of the correlation. Blue edges = positive correlations. Node abbreviations: D1: Bothered by things, D2: Had trouble keeping in mind, D3: Felt depressed, D4: Everything an effort, D5: Hopelessness, D6: Felt fear, D7: Sleep was restless, D8: Lack of happiness, D9: Felt lonely, D10: Could not get going.

**Appendix Table 1.** Edge weights of the cross-sectional networks of depressive symptoms in Wave 1 and Wave 2

|  | **D1** | **D2** | **D3** | **D4** | **D5** | **D6** | **D7** | **D8** | **D9** | **D10** |
| --- | --- | --- | --- | --- | --- | --- | --- | --- | --- | --- |
| **D1** | - | 0.19 | 0.30 | 0.08 | -0.02 | 0.06 | 0.07 | 0.09 | 0.05 | 0.06 |
| **D2** | 0.20 | - | 0.22 | 0.16 | -0.01 | 0.05 | 0.05 | 0.01 | 0.04 | 0.04 |
| **D3** | 0.26 | 0.24 | - | 0.23 | -0.02 | 0.07 | 0.07 | 0.08 | 0.10 | 0.07 |
| **D4** | 0.08 | 0.19 | 0.27 | - | 0.00 | 0.08 | 0.10 | 0.04 | 0.07 | 0.13 |
| **D5** | -0.02 | -0.06 | -0.03 | 0.00 | - | -0.03 | 0.00 | **0.33** | 0.01 | 0.01 |
| **D6** | 0.07 | 0.04 | 0.07 | 0.08 | -0.03 | - | 0.08 | 0.03 | 0.12 | 0.22 |
| **D7** | 0.10 | 0.05 | 0.07 | 0.09 | 0.00 | 0.13 | - | 0.07 | 0.08 | 0.05 |
| **D8** | 0.08 | 0.00 | 0.06 | 0.02 | 0.35 | 0.02 | 0.02 | - | 0.11 | 0.04 |
| **D9** | 0.05 | 0.02 | 0.10 | 0.06 | 0.00 | 0.15 | 0.09 | 0.07 | - | 0.27 |
| **D10** | 0.05 | 0.04 | 0.09 | 0.09 | 0.00 | 0.18 | 0.05 | 0.05 | 0.32 | - |

Note: Edge weights of the cross-sectional network in Wave 1 are shown above the diagonal, while the edge weights of the network in Wave 2 are displayed below the diagonal.

D1: Bothered by things, D2: Had trouble keeping in mind, D3: Felt depressed, D4: Everything an effort, D5: Hopelessness, D6: Felt fear, D7: Sleep was restless, D8: Lack of happiness, D9: Felt lonely, D10: Could not get going.

**Appendix Table 2.** Centrality indices in Wave 1 and Wave 2

|  | **Wave 1** | | |  | **Wave 2** | | |
| --- | --- | --- | --- | --- | --- | --- | --- |
|  | **Betweenness** | **Closeness** | **Strength** |  | **Betweenness** | **Closeness** | **Strength** |
| **D1** | -0.385 | 0.684 | 0.626 |  | 1.630 | 1.227 | 0.499 |
| **D2** | -1.026 | -0.319 | -0.111 |  | -0.815 | 0.167 | 0.168 |
| **D3** | 0.897 | 1.361 | 1.768 |  | 1.358 | 1.192 | 1.943 |
| **D4** | 0.577 | 0.915 | 0.485 |  | -0.543 | 0.526 | 0.361 |
| **D5** | -1.026 | -1.546 | -1.856 |  | -0.815 | -1.785 | -1.687 |
| **D6** | -1.026 | -0.896 | -0.341 |  | -0.543 | -0.405 | -0.166 |
| **D7** | -1.026 | -1.181 | -1.186 |  | -0.815 | -0.710 | -1.110 |
| **D8** | 1.539 | -0.347 | -0.041 |  | 1.086 | -1.154 | -0.689 |
| **D9** | 0.577 | 0.939 | 0.169 |  | 0.272 | 0.734 | 0.299 |
| **D10** | 0.897 | 0.390 | 0.487 |  | -0.815 | 0.208 | 0.383 |

D1: Bothered by things, D2: Had trouble keeping in mind, D3: Felt depressed, D4: Everything an effort, D5: Hopelessness, D6: Felt fear, D7: Sleep was restless, D8: Lack of happiness, D9: Felt lonely, D10: Could not get going.

**
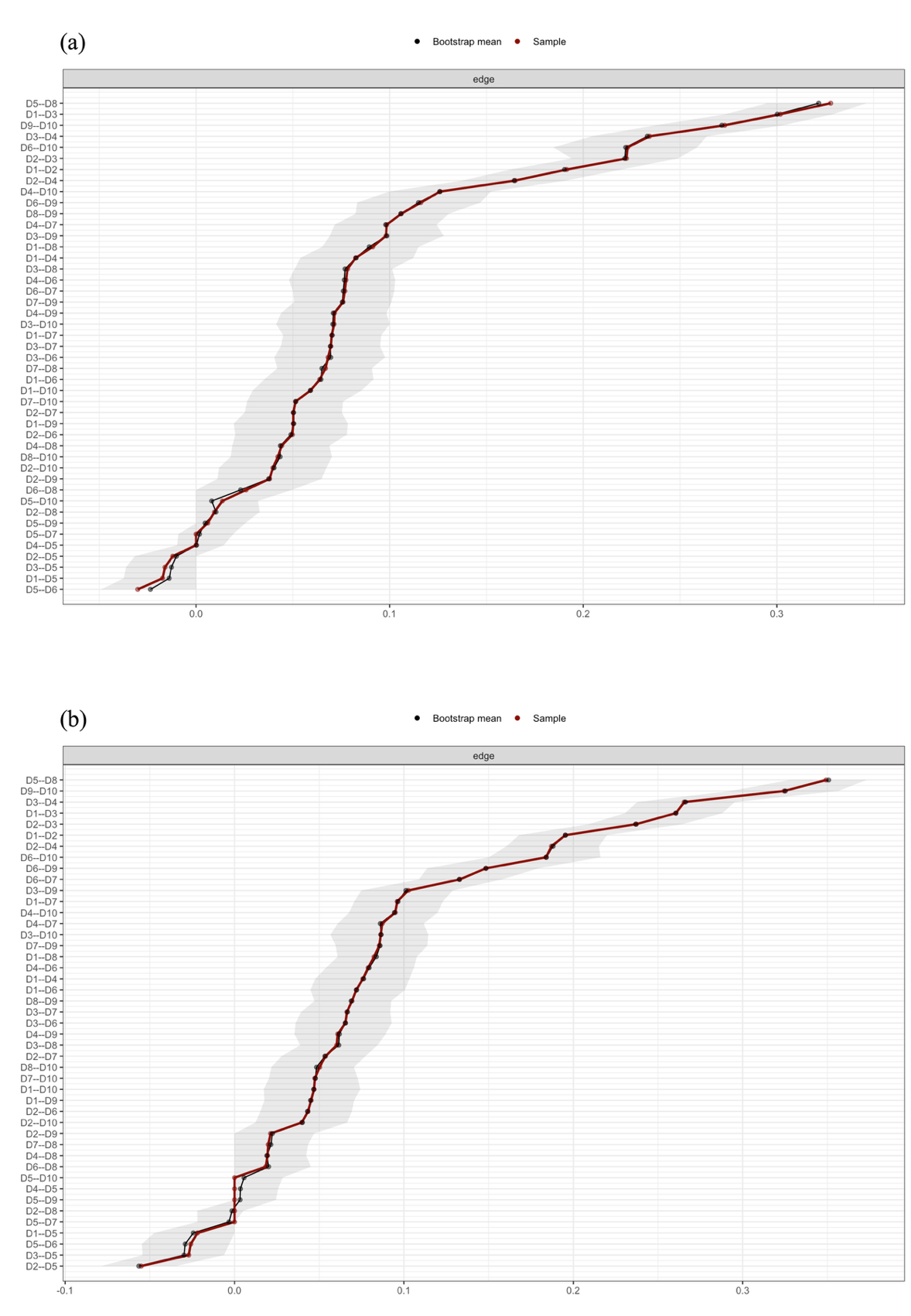
Appendix Figure 2.** Accuracy of edge weights of cross-sectional networks of depressive symptoms in Wave 1 (a) and Wave 2 (b). The red lines indicate edge weights from our samples. Black lines are edge weights generated based on 1000 random bootstrap samples. Consistency between red lines and black lines suggests high accuracy. Grey areas are bootstrap confidence intervals (CI), with narrower intervals indicating higher precision. If the red line falls within the grey CI, the edge is considered stable. The X-axis represents the effect size value between two nodes. The Y-axis represents the combination of two nodes in the cross-sectional network.

**
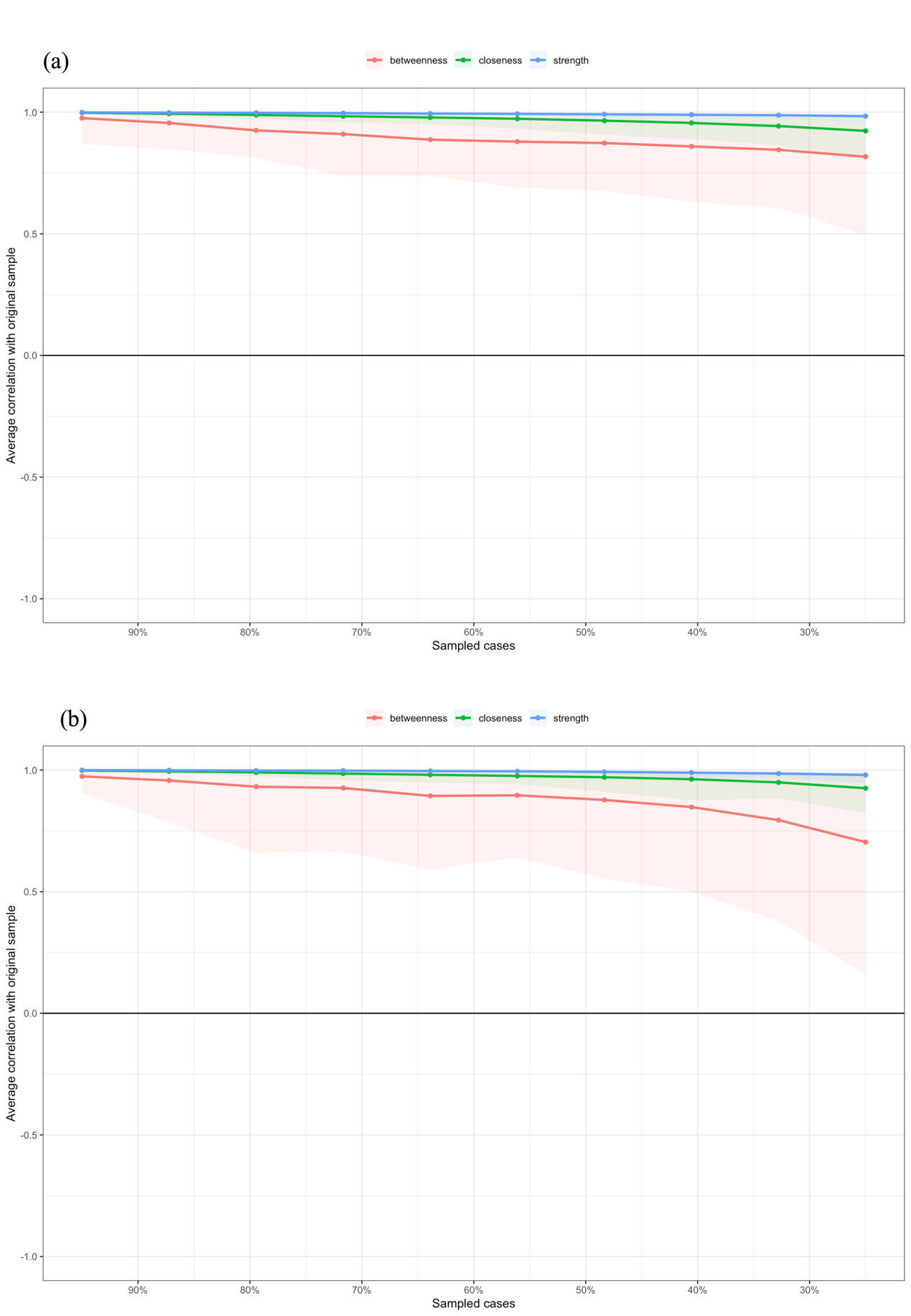
Appendix Figure 3.** Stability of centrality indices for cross-sectional networks of depressive symptoms in Wave 1 (a) and Wave 2 (b). Lines link the means of correlations from subsets with an increasing number of excluded participants. Colored areas indicate the range of correlations from the 2.5th to the 97.5th quantile.

**
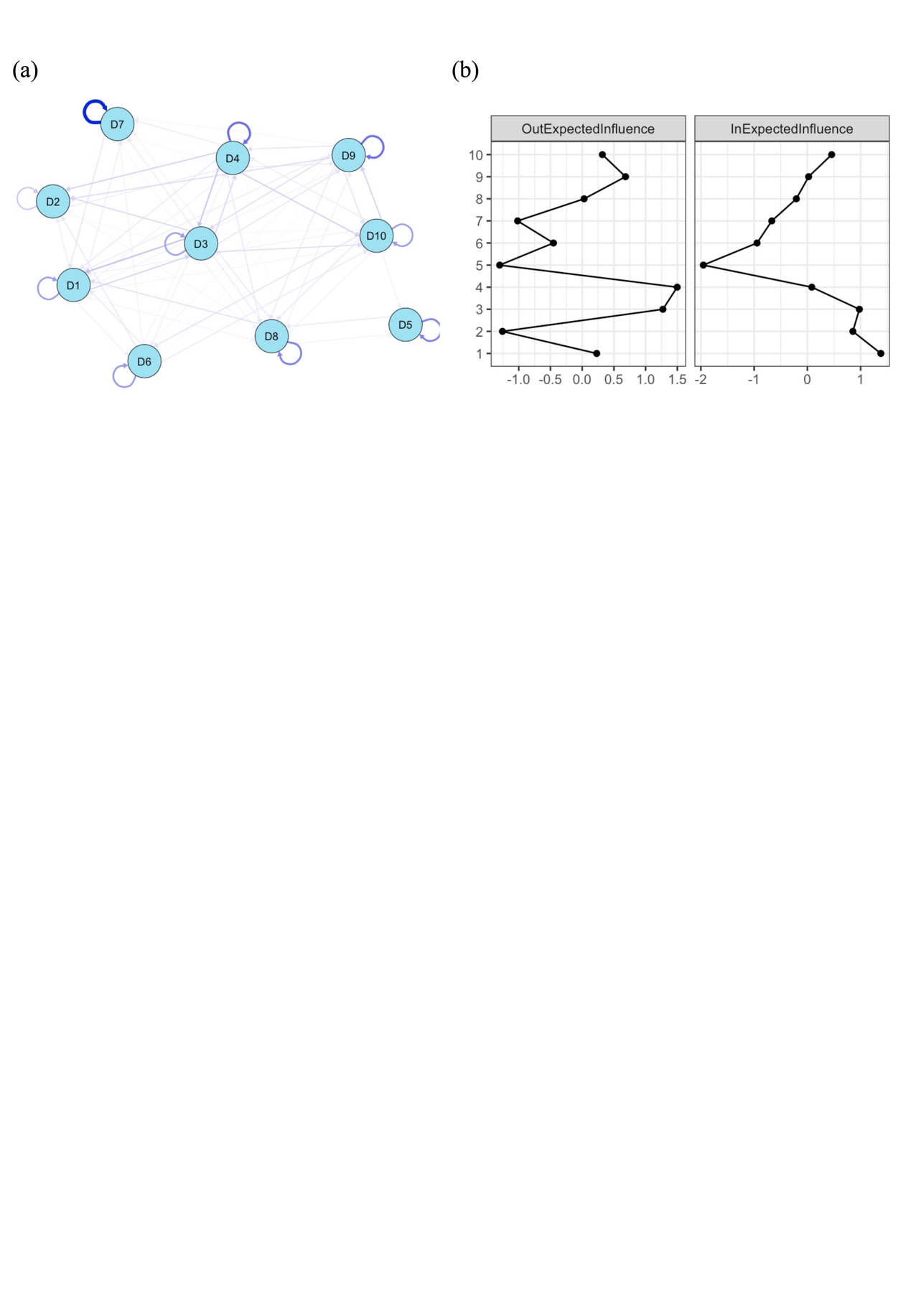
Appendix Figure 4.** The longitudinal network (a) and standardized centrality indices (b) of depressive symptoms between Wave 1 and Wave 2. The thickness of the edges represents the magnitude of the correlation. Blue edges = positive correlations. The arrows on the lines show the predictive effects of depressive symptoms in Wave 1 on symptoms in Wave 2. Loop arrows represent autoregressive effects. Node abbreviations: D1: Bothered by things, D2: Had trouble keeping in mind, D3: Felt depressed, D4: Everything an effort, D5: Hopelessness, D6: Felt fear, D7: Sleep was restless, D8: Lack of happiness, D9: Felt lonely, D10: Could not get going.

**Appendix Table 3.** Edge weights of a dynamic network of depressive symptoms, from Wave 1 (column) to Wave 2 (row)

|  | **D1** | **D2** | **D3** | **D4** | **D5** | **D6** | **D7** | **D8** | **D9** | **D10** |
| --- | --- | --- | --- | --- | --- | --- | --- | --- | --- | --- |
| **D1** | 0.14 | 0.03 | 0.06 | 0.02 | 0.00 | 0.02 | 0.03 | 0.02 | 0.02 | 0.02 |
| **D2** | 0.00 | 0.08 | 0.01 | 0.03 | 0.00 | 0.00 | 0.00 | 0.00 | 0.00 | 0.00 |
| **D3** | 0.09 | 0.06 | 0.13 | 0.06 | 0.00 | 0.02 | 0.02 | 0.02 | 0.04 | 0.05 |
| **D4** | 0.04 | 0.07 | 0.08 | 0.20 | 0.00 | 0.02 | 0.03 | 0.04 | 0.03 | 0.07 |
| **D5** | 0.00 | 0.00 | 0.00 | 0.00 | 0.17 | 0.00 | 0.00 | 0.03 | 0.00 | 0.00 |
| **D6** | 0.03 | 0.04 | 0.00 | 0.00 | 0.00 | 0.14 | 0.02 | 0.00 | 0.01 | 0.03 |
| **D7** | 0.04 | 0.00 | 0.01 | 0.01 | 0.00 | 0.00 | 0.35 | 0.00 | 0.00 | 0.00 |
| **D8** | 0.05 | 0.01 | 0.04 | 0.00 | 0.02 | 0.00 | 0.03 | 0.17 | 0.03 | 0.02 |
| **D9** | 0.03 | 0.05 | 0.05 | 0.04 | 0.00 | 0.01 | 0.01 | 0.03 | 0.20 | 0.04 |
| **D10** | 0.02 | 0.00 | 0.01 | 0.03 | 0.02 | 0.04 | 0.00 | 0.04 | 0.07 | 0.14 |

D1: Bothered by things, D2: Had trouble keeping in mind, D3: Felt depressed, D4: Everything an effort, D5: Hopelessness, D6: Felt fear, D7: Sleep was restless, D8: Lack of happiness, D9: Felt lonely, D10: Could not get going.

**Appendix Table 4.** In-Expected Influence and Out-Expected Influence in the longitudinal network

|  | **In-Expected Influence** | **Out-Expected Influence** |
| --- | --- | --- |
| **D1** | 1.380 | 0.229 |
| **D2** | 0.854 | -1.257 |
| **D3** | 0.977 | 1.272 |
| **D4** | 0.083 | 1.498 |
| **D5** | -1.953 | -1.301 |
| **D6** | -0.945 | -0.453 |
| **D7** | -0.668 | -1.019 |
| **D8** | -0.207 | 0.030 |
| **D9** | 0.023 | 0.683 |
| **D10** | 0.456 | 0.318 |

D1: Bothered by things, D2: Had trouble keeping in mind, D3: Felt depressed, D4: Everything an effort, D5: Hopelessness, D6: Felt fear, D7: Sleep was restless, D8: Lack of happiness, D9: Felt lonely, D10: Could not get going.

**
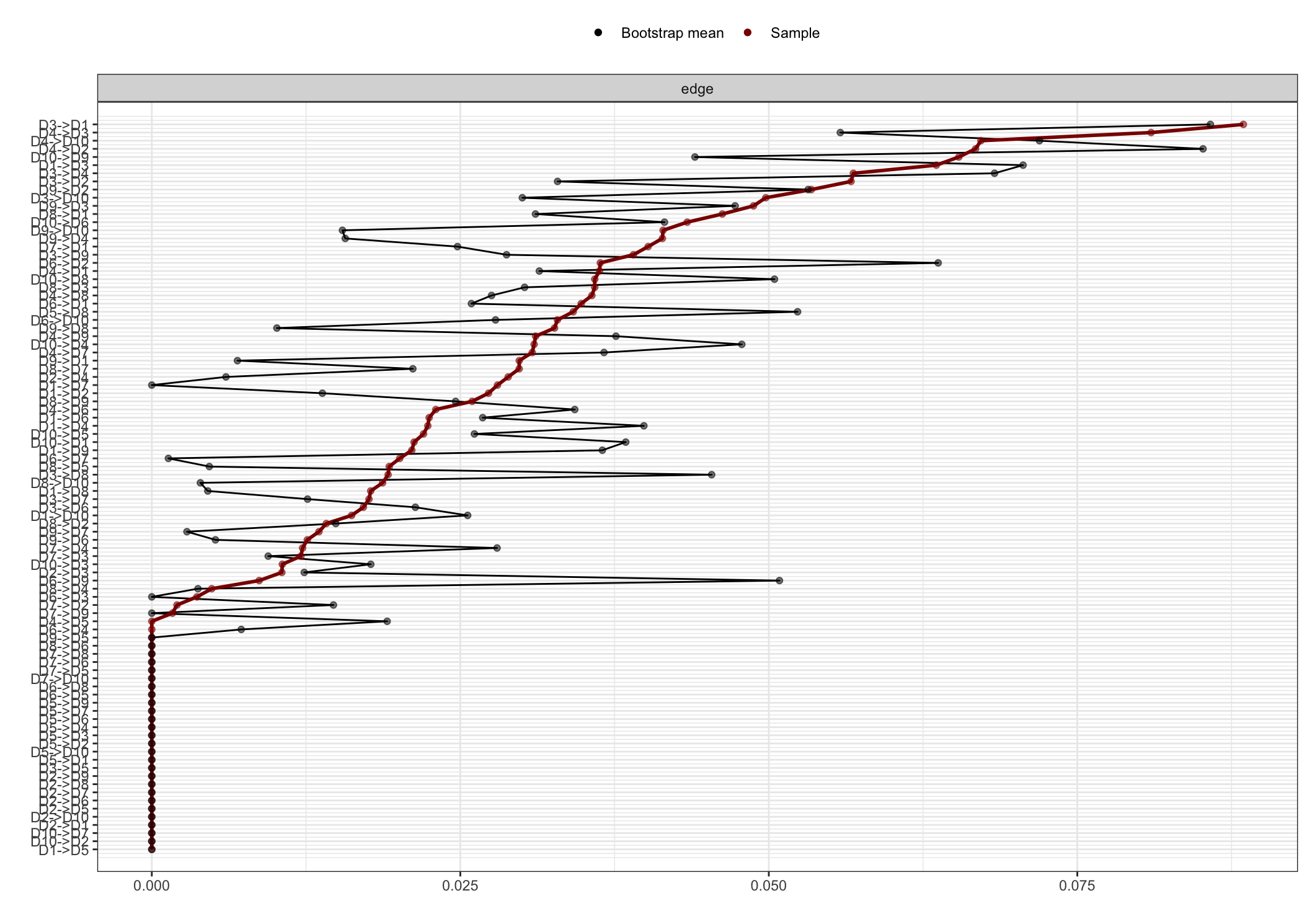
Appendix Figure 5.** Accuracy of edge weights in the dynamic network of depressive symptoms. Red dots and lines are the edge weights from the sample. Black dots and lines are edge weights from 1000 random bootstrap samples.
